# Frenulate siboglinids at high Arctic methane seeps and insight into high latitude frenulate distribution

**DOI:** 10.1002/ece3.5988

**Published:** 2020-01-09

**Authors:** Arunima Sen, Alena Didriksen, Stéphane Hourdez, Mette Marianne Svenning, Tine L. Rasmussen

**Affiliations:** ^1^ CAGE—Centre for Arctic Gas Hydrate, Environment and Climate Department of Geosciences UiT The Arctic University of Norway Tromsø Norway; ^2^ Department of Arctic and Marine Biology UiT The Arctic University of Norway Tromsø Norway; ^3^ Laboratoire d'écogéochimie des Environnements Benthiques UMR8222 CNRS‐Sorbonne Université Banyuls‐sur‐Mer France; ^4^ Present address: Faculty of Biosciences and Aquaculture Nord University Bodø Norway

**Keywords:** Atlantic currents, dispersal, Fram Strait, gas seepage, larvae, *Oligobrachia*, *Oligobrachia* juveniles, Vestnesa

## Abstract

Frenulate species were identified from a high Arctic methane seep area on Vestnesa Ridge, western Svalbard margin (79°N, Fram Strait) based on mitochondrial cytochrome oxidase subunit I (mtCOI). Two species were found: *Oligobrachia haakonmosbiensis*, and a new, distinct, and undescribed *Oligobrachia* species. The new species adds to the cryptic *Oligobrachia* species complex found at high latitude methane seeps in the north Atlantic and the Arctic. However, this species displays a curled tube morphology and light brown coloration that could serve to distinguish it from other members of the complex. A number of single tentacle individuals were recovered which were initially thought to be members of the only unitentaculate genus, *Siboglinum*. However, sequencing revealed them to be the new species and the single tentacle morphology, in addition to thin, colorless, and ringless tubes indicate that they are juveniles. This is the first known report of juveniles of northern *Oligobrachia*. Since the juveniles all appeared to be at about the same developmental stage, it is possible that reproduction is either synchronized within the species, or that despite continuous reproduction, settlement, and growth in the sediment only takes place at specific periods. The new find of the well‐known species *O. haakonmosbiensis* extends its range from the Norwegian Sea to high latitudes of the Arctic in the Fram Strait. We suggest bottom currents serve as the main distribution mechanism for high latitude *Oligobrachia* species and that water depth constitutes a major dispersal barrier. This explains the lack of overlap between the distributions of northern *Oligobrachia* species despite exposure to similar current regimes. Our results point toward a single speciation event within the *Oligobrachia* clade, and we suggest that this occurred in the late Neogene, when topographical changes occurred and exchanges between Arctic and North Atlantic water masses and subsequent thermohaline circulation intensified.

## Graphical abstract

The deepwater (~1,200 m depth), high Arctic (79°N) Vestnesa seep contains two frenulate species: *Oligobrachia haakonmosbiensis* and a potentially new species. Multitentaculate adults and unitentaculate juveniles of the new species were present. We propose that bottom currents serve as the main dispersal mechanism for high latitude frenulates, although water depth functions as a major distributional barrier.

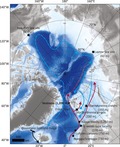

## 1 INTRODUCTION

**Figure 1:**
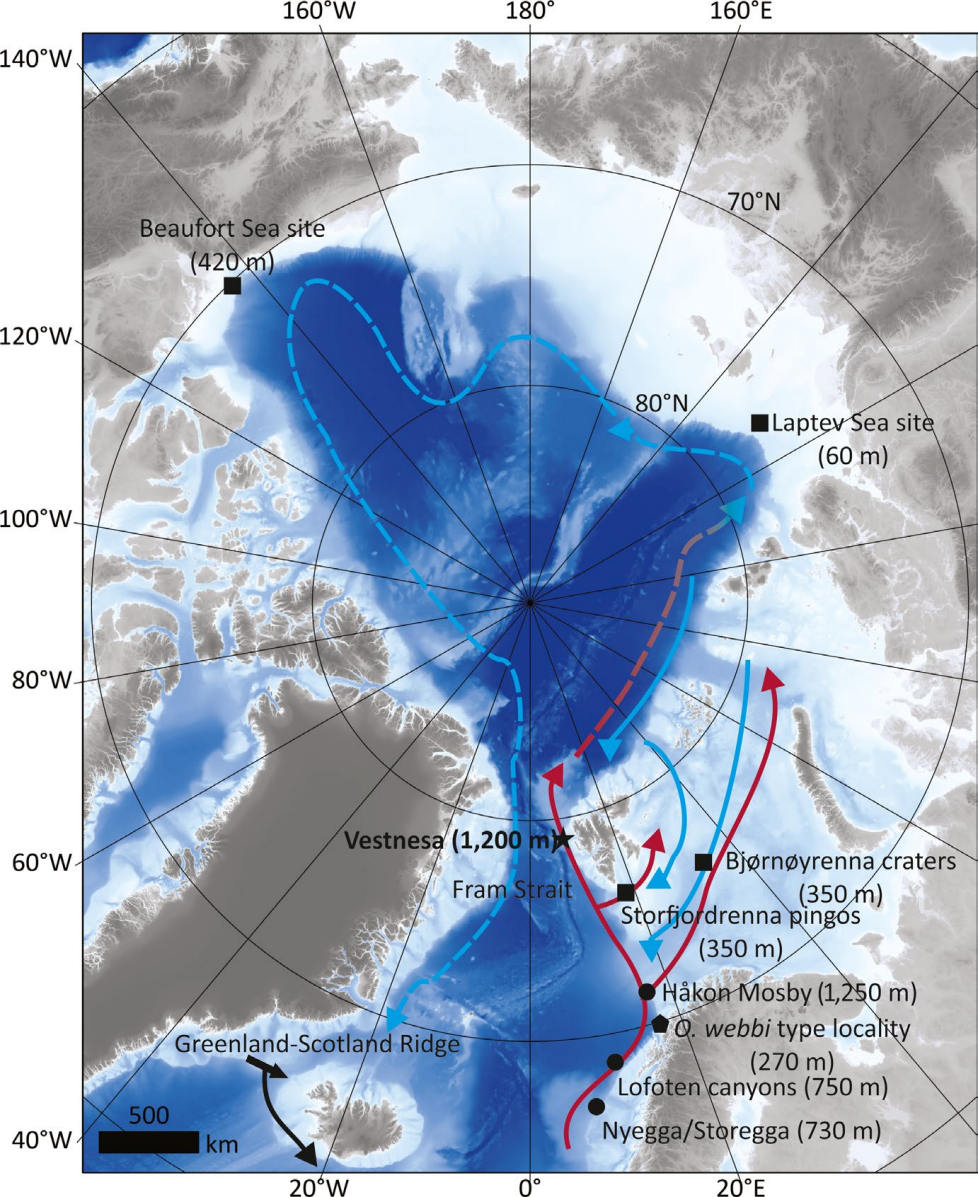
Map of the Arctic Ocean and fringing shelf seas with known seep sites. Type locality of *Oligobrachia webbi* is marked with a pentagon. Dots represent Norwegian Sea seep sites hosting *O. haakonmosbiensis*: Storegga/Nyegga, Lofoten canyon site, and Håkon Mosby mud volcano. Squares represent Arctic sites hosting the *Oligobrachia* sp. CPL‐clade: Storfjordrenna “pingos” and Bjørnøyrenna “craters” in the Barents Sea, the Laptev Sea site, and a mud volcano in the Canadian Beaufort Sea. The study location of Vestnesa in the Fram Strait, between Greenland and Svalbard, is marked with a star. Approximate water depths of all sites are shown in parentheses. The Greenland–Scotland Ridge, which separates the Nordic seas from the North Atlantic Ocean, is located at about 500–800 m water depth underlies and connects Iceland, the Faroe Islands, and Scotland (continues beyond the field of view of the map). A simplified representation of the main water currents are displayed with arrows. Red represents warm Atlantic water, and blue represents cold, Arctic/Polar water. Solid lines indicate surface water, and stippled lines represent subsurface water. Bathymetry for this map was obtained from IBCAO (Jakobsson et al., 2012)

Frenulate siboglinid worms are the dominant fauna of cold seeps in northern latitude regions (Åstrom, Carroll, Ambrose, & Carroll, 2016; Åström et al., 2018; Decker et al., 2012, p. 1; Decker & Olu, 2012; Gebruk et al., 2003; Hovland & Svensen, 2006; Paull et al., 2015; Rybakova (Goroslavskaya), Galkin, Bergmann, Soltwedel, and Gebruk, 2013; Savvichev et al., 2018; Sen et al., 2019; Sen, Åström, et al., 2018a; Sen, Duperron, et al., 2018b). They are also the only confirmed chemosynthesis‐based animals of the communities at nearly all high latitude seeps (Sen, Åström, et al., 2018a; Sen, Duperron, et al., 2018b) and therefore integral to the functioning of these ecosystems. Currently, based on mitochondrial COI gene sequences, two putative species of cryptic frenulates are known to exist across high latitude Atlantic and Arctic seeps. These are *Oligobrachia haakonmosbiensis* (found at seep sites in the Norwegian Sea such as the Håkon Mosby mud volcano, the Nyegga/Storegga slide, and a canyon site off the Lofoten islands of northern Norway (Sen, Duperron, et al., 2018b; Smirnov, 2014, 2000), and *Oligobrachia* sp. CPL‐clade found in the Barents Sea, the Laptev Sea and the Canadian Beaufort Sea (Lee et al., 2019; Sen, Duperron, et al., 2018b) (Figure 1). It should further be noted that either of these species could correspond with the species *Oligobrachia webbi* found off the coast of Tromsø, northern Norway (Brattegard, 1966). This species is similar in morphology to both *O. haakonmosbiensis* and the CPL‐clade members, but the lack of both sequences and DNA‐extractable tissue has prevented molecular‐based identification (Sen, Duperron, et al., 2018b). Therefore, high latitude seep frenulate species identifications presently exist for two distinct species: *O. haakonmosbiensis* and *Oligobrachia* sp. CPL‐clade. The distribution patterns of these species appear to follow a simple north–south trend, with *O. haakonmosbiensis* occurring in the more southern sites (Norwegian Sea/high latitude north Atlantic), and the CPL‐clade members inhabiting the more northern, Arctic sites (Sen, Duperron, et al., 2018b) (Figure 1). Additionally, all the southern, *O. haakonmosbiensis* sites are found on the slope at depths of 750 m to 1,250 m, while all the CPL‐clade members so far are found at sites on the shelf, at depths of 60–420 m. Thus, examining frenulate species at a site that combines the two variables of latitude and water depth could shed light on the biogeography of the species. Specifically, an examination of frenulate species at northern, or high Arctic sites that are also located at water depths comparable to the southern slope sites, could be insightful.

The Vestnesa Ridge on the western Svalbard margin in the Fram Strait contains a number of pockmarks indicative of methane seepage (Bünz, Polyanov, Vadakkepuliyambatta, Consolaro, & Mienert, 2012; Plaza‐Faverola et al., 2015; Vogt, Crane, Sundvor, Max, & Pfirman, 1994). The active sites are located at 79°N at a water depth of approximately 1,200 m. Visual surveys and sample collections have demonstrated that these sites, similar to other high latitude seeps, are dominated by frenulate worms, though species identification has not been conducted (Åström et al., 2018). From a geological perspective, Vestnesa is significant; it is close to an ultraslow spreading center where hydrothermal vents exist (Pedersen et al., 2010; Sweetman, Levin, Rapp, & Schander, 2013), and tectonic stress from rifting of ridges and shear motion from transform faults appear to modulate seafloor gas release (Plaza‐Faverola et al., 2015). Therefore, Vestnesa has been intensively studied from a geological and geophysical perspective (e.g., Bünz et al., 2012; Consolaro et al., 2015; Hansen, Hoff, Sztybor, & Rasmussen, 2017; Plaza‐Faverola et al., 2015; Singhroha, Bünz, Plaza‐Faverola, & Chand, 2016; Sztybor & Rasmussen, 2017), while biological studies of living faunas are limited (e.g., Åström et al., 2018). Any higher‐order understanding of the ecology of Vestnesa seeps requires knowledge of the dominant community members, particularly since frenulates can be considered ecosystem engineers due to their influence on the biology as well as the physical characteristics of their habitats (Dando, Southward, Southward, Lamont, & Harvey, 2008; Sen, Åström, et al., 2018a; Sen et al., 2019). Furthermore, identifying frenulate species at the relatively deep (i.e., slope) and high Arctic (79°N) area that Vestnesa is located in would shed light on the large‐scale patterns of the distribution of high latitude chemosynthesis‐based seep animals.

In this study, we used mitochondrial cytochrome oxidase subunit I (mtCOI) sequencing to identify frenulates from pockmarks on the high Arctic, Vestnesa Ridge. This gene has been used widely for distinguishing species within the siboglinid clade, including frenulates (Braby, Rouse, Johnson, Jones, & Vrijenhoek, 2007; Chevaldonné, Jollivet, Desbruyères, Lutz, & Vrijenhoek, 2002; Feldman et al., 1998; Fujikura, Fujiwara, & Kawato, 2006; Hilário, Johnson, Cunha, & Vrijenhoek, 2010; Jones, Johnson, Rouse, & Vrijenhoek, 2008; Kojima et al., 2002). We provide information on the identity of the species of frenulates that constitute the most dominant members of Vestnesa faunal communities and how certain oceanographic processes may affect the distribution and speciation of cold‐seep frenulates at high latitudes.

## 2 MATERIALS AND METHODS

**Figure 2:**
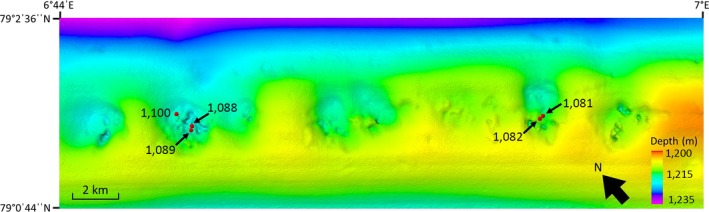
Bathymetric map of section of the Vestnesa ridge, where numerous pockmarks are visible. Locations of box cores from which frenulate worms were collected are shown with red dots with corresponding box core numbers indicated

Frenulate samples were collected for this study in July 2018 during a cruise with the R/V *Helmer Hanssen* (UiT, The Arctic University of Norway) to the crest of Vestnesa Ridge where a series of pockmarks have been observed (Figure 2). Gas flares rising into the water column have been recorded on echosounders from a number of these pockmarks, though detailed investigations and research has been carried out largely at a smaller subset of these pockmarks (e.g., Åström et al., 2018; Hansen et al., 2017; Sztybor & Rasmussen, 2017). Two pockmarks were targeted for this study (Figure 2) since frenulates have previously been observed in dense aggregations within them (Åström et al., 2018; Sztybor & Rasmussen, 2017). Box cores were deployed from the ship at a few different locations within these pockmarks (Figure 2). On deck, sediment containing frenulate worms was rinsed and the individual worms were removed. Worms were kept in chilled, filtered seawater, in the dark, until they were processed.

Individuals were carefully extracted from their tubes under a dissecting microscope, with the help of fine forceps and a paintbrush, and the type of tube was noted in each case. Worms were not extracted from their tubes if they and/or their tubes were very fragile since this led to the dissolution of the worms and destruction of the tubes. Upon extraction, tissues were stored immediately in absolute ethanol. Worms within delicate tubes were stored in absolute ethanol with the tubes intact.

In the laboratory, DNA was extracted from worm samples using the DNEasy Blood and Tissue kit (Qiagen), following the manufacturer's instructions. DNA concentrations for each sample were checked and measured through gel electrophoresis and a microphotometer (Nanodrop). A fragment of mtCOI was amplified, purified and sequenced. The “universal” primers LCOI‐1490 and HCOI‐2198 were used for the barcode approach (Folmer, Black, Hoeh, Lutz, & Vrijenhoek, 1994) (35 cycles at 94°C for 1 min; 52°C for 1 min and 72°C for 1 min). Positive amplification was checked on an agarose gel (HiYield Gel; RBC Bioscience), and PCR products were isolated using a PCR DNA Extraction and Cleanup Kit (RBC Bioscience). Sequencing was conducted at the DNA sequencing core facility at the Medical Genetics Department of the University Hospital of North Norway.

The sequences were trimmed and manually checked with Codon Code Aligner. The sequences acquired in this study as well as published sequences (Lösekann et al., 2008; Sen, Duperron, et al., 2018b) were aligned with a Clustal algorithm. The maximum likelihood (ML) phylogenetic tree was produced with PhyML, under a general time‐reversible model with variable evolutionary rates among sites (gamma distribution) and invariant sites. Only likelihood values higher than 0.80 are reported on the tree. Sequences corresponding to the *Oligobrachia* clade (see results) were used for automatic barcode gap discovery (ABGD) (Puillandre, Lambert, Brouillet, & Achaz, 2012). The haplotype network for the *Oligobrachia* sequences was obtained and edited with PopART (Leigh & Bryant, 2015) with a TCS approach (Clement, Posada, & Crandall, 2000). The sequences were provided with location as a trait to allow a representation with geography as a possible underlying factor.

## 3 RESULTS

**Figure 3:**
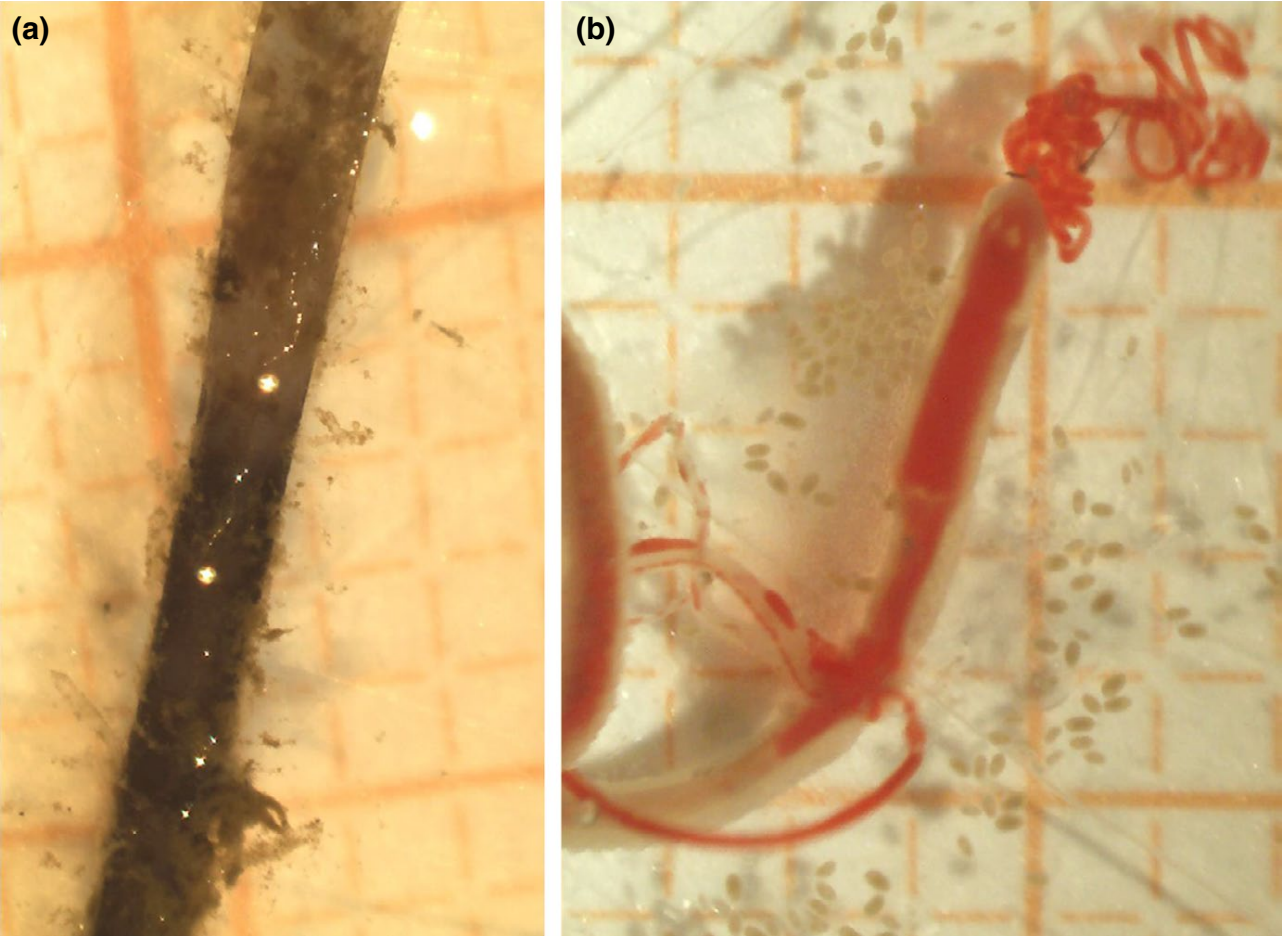
Specimens of *Oligobrachia haakonmosbiensis*. A: Anterior part of tubes showing the fairly straight tube and dark brown color. B: Image of live individual immediately after extraction from its tube. The image is taken from the ventral side, but tentacles are clearly visible. Note presence of embryos and larvae spilled out from the tube during extraction. In both panels, millimeter paper is used for scale: each square is 1 × 1 mm

**Figure 4:**
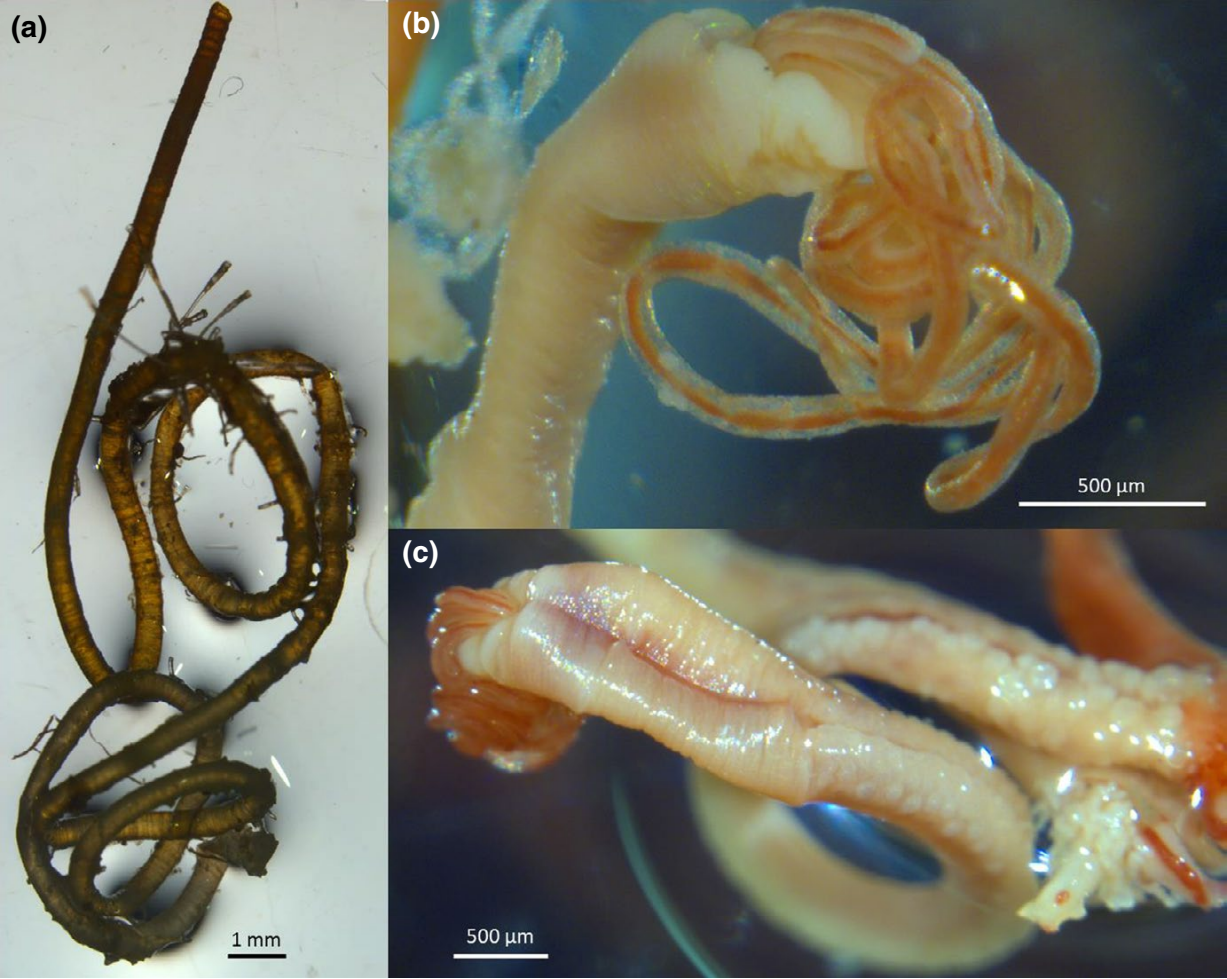
Adult specimens of the possible new Vestnesa species. (a) Anterior portion of the yellow‐brown curled tube (note presence of epibionts). (b) Lateral, close‐up view of forepart and cephalic lobe of an adult worm extracted from the tube. (c) Dorsal view of the same individual, with forepart and zone of papillae clearly visible. Images were taken postfixation in ethanol; therefore, coloration and relative body sizes are not necessarily representative (i.e., discoloration, shrinking and/or swelling might have occurred)

**Figure 5:**
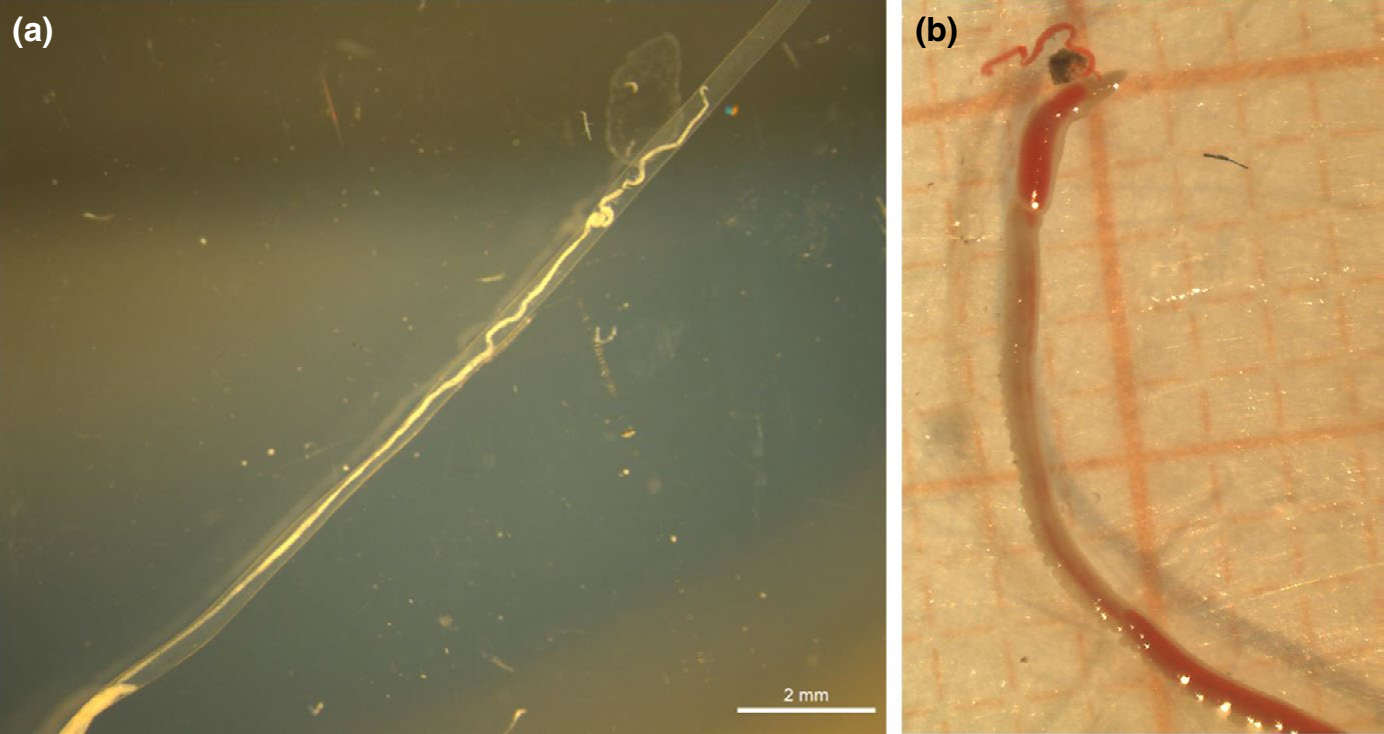
Juvenile specimens of the new Vestnesa species. (a) Juvenile individual preserved in ethanol within its thin tube. Lack of color is due to the preservation. Note the single, long tentacle and the extremely thin, colorless, and ringless tube. (b) Live juvenile individual extracted from its tube. A single tentacle is visible; however, this tentacle was broken off and does not represent its entire length. The squares on the millimeter paper in the background are 1 × 1 mm

Three distinct types of tubes were found: brown‐black straight tubes, typical of *Oligobrachia* species (Sen, Duperron, et al., 2018b; Smirnov, 2000), lighter brown, curled tube, somewhat reminiscent of *Sclerolinum* tubes (Smirnov, 2000) (Figures 3 and 4), and straight, transparent and colorless tubes that appeared to be thinner and more fragile than the others (Figure 5). The worms within the straight, dark tubes resemble other known northern seep *Oligobrachia* species (nonpinnule bearing, multiple tentacles) (Figure 3). This was also the morphotype seen within the brown, curled tubes (Figure 4). The worms within the thin, transparent tubes had a much more distinct appearance: They contained only a single tentacle and were initially thought to be members of the only unitentaculate genus, *Siboglinum* (Figure 5).

**Figure 6:**
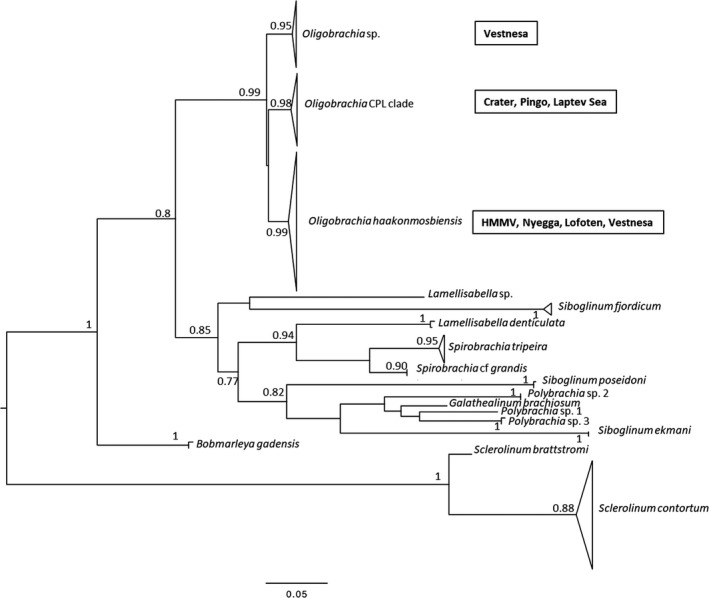
Maximum likelihood (ML) tree obtained with PhyML on a 473‐bp alignment of a fragment of the mitochondrial COI gene for frenulate Siboglinidae, with a SPR‐tree‐searching approach. The moniliferan siboglinid *Sclerolinum* spp. was used as the outgroup. Branch support:approximate likelihood ratio test (aLRT). Nodes with aLRT values greater than 0.8 are included or shown. Accession numbers: see Table 1 and Sen, Duperron, et al., 2018b

With respect to the results from the molecular methods, the tree obtained from the alignment is relatively robust (Figure 6). A well‐constrained clade (ML = 0.99) contains all the *Oligobrachia* sequences. This lineage then splits into three *Oligobrachia* clusters, each supported by high likelihood values (0.98–0.99). The relationships between these three clades remain poorly resolved, suggesting a rapid (and possibly concomitant) radiation of all three lineages. The tree reveals the presence of two distinct species among the Vestnesa specimens (Figure 6), and both species are members of the *Oligobrachia* clade. One type of sequence corresponds to *Oligobrachia haakonmosbiensis*, extending the range of this species to the high Arctic. All individuals in straight, dark tubes belong to this species. The second type of *Oligobrachia* sequence was distinct from both *O. haakonmosbiensis* and *Oligobrachia* sp. CPL‐clade (the two *Oligobrachia* species for which sequences are available) and therefore could represent a new species altogether. Individuals in the light brown, curled tubes as well as the individuals in the straight, thin and transparent tubes have sequences corresponding to this potentially new species. The putative new species displays two very distinct morphotypes, in terms of both tube morphology and soft tissue structures. The multitentaculate morphology is shown in Figure 4, and the unitentaculate morphology, at least of the anterior parts, is shown in Figure 5. Live images were nearly impossible to take due to rough sea conditions. As a result, detailed images were not obtained during the cruise itself. After sequencing, samples were not adequate to allow for full taxonomic descriptions; therefore, we discuss the new species based on sequences and overall morphology.

**Figure 7:**
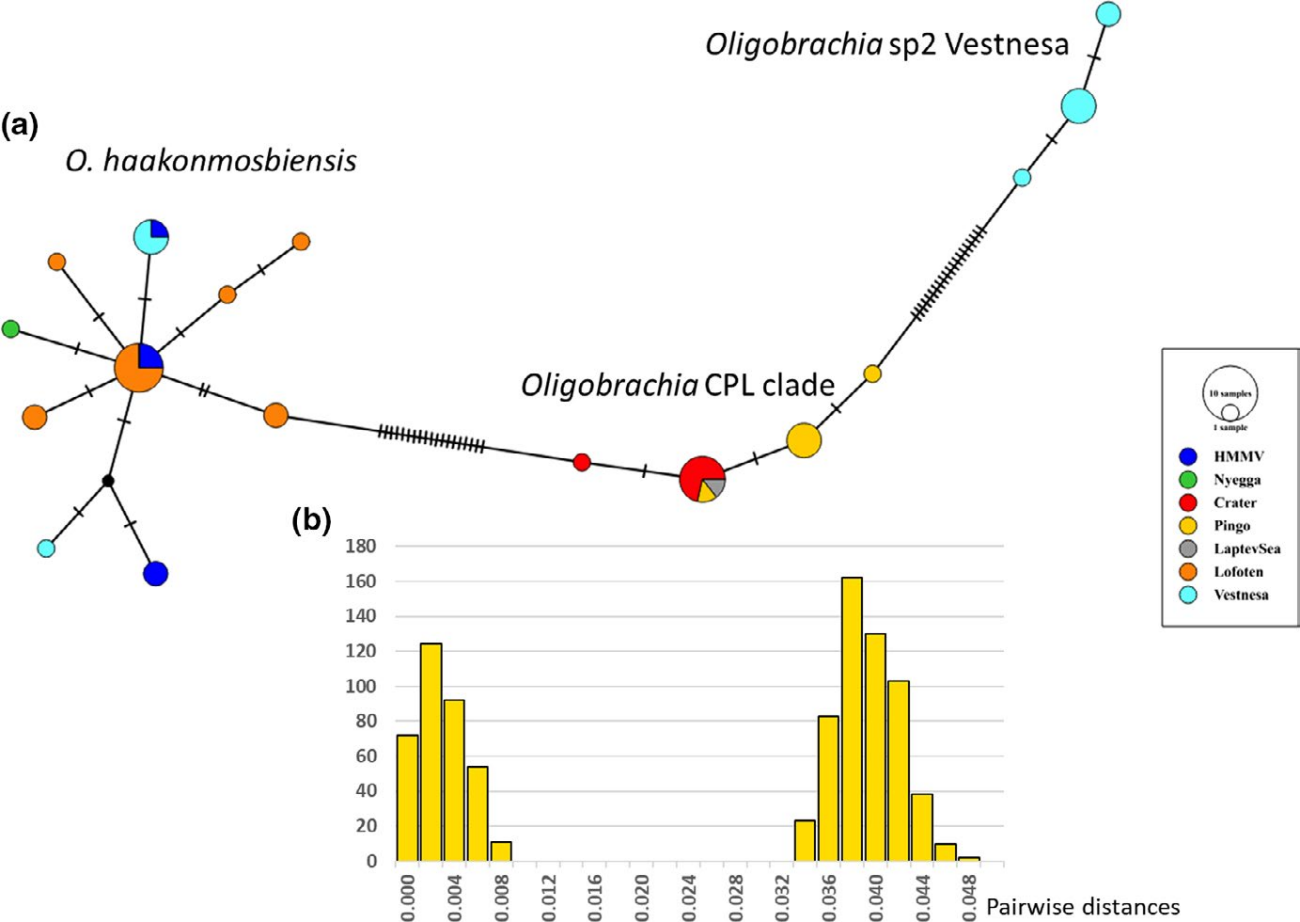
Haplotype network and barcode gap detection. (a) Haplotype network obtained with a TCS methods in PopART for COI sequences (*n* = 43) in the *Oligobrachia* spp. clade (see Figure 3). *Oligobrachia* sp2 Vestnesa refers to the new sequences obtained in this study. Tick marks indicate mutational steps. (b) Automatic barcode gap discovery (ABGD) pairwise distance distribution histogram showing two groups of sequences (prior maximal distance *p* = .00167)

The haplotype network established with all the available *Oligobrachia* sequences also reveals the presence of 3 groups of sequences (Figure 7). The *O. haakonmosbiensis* cluster (including some sequences from Vestnesa) differs from the *Oligobrachia* CPL‐clade by 19 mutations, and the latter group differs from the second new Vestnesa species by 20 mutations. The presence of these three groups of sequences (=species) is also supported by automatic barcode gap discovery (ABGD; Figure 5a; *p* = .00169). A single gap appears, separating the within‐clade distance values (0.24 ± 0.18%) and the between‐clade distance values (3.86 ± 0.24%). Based on 0.5% per million years mutation rates established for hydrothermal vent species (including siboglinids; Chevaldonné et al., 2002), this would correspond to about 7.7 million years of divergence.

## 4 DISCUSSION

### 4.1 *Oligobrachia* species at Vestnesa and other high latitude seeps

The results of the molecular analyses demonstrate that the sampled pockmarks at Vestnesa Ridge contain two species of *Oligobrachia*. The presence of *Oligobrachia* overall is not surprising, since during sampling, the worms were seen to resemble this cryptic species complex based on overall body morphology (Sen, Duperron, et al., 2018b). The more pertinent question was regarding the individual species itself. The deeper, slope species, *Oligobrachia haakonmosbiens,* found presently at seeps in the Norwegian Sea, and/or, the shallower shelf species, *Oligobrachia* sp. CPL‐clade, found in high Arctic locations in the Barents and Laptev Seas (Sen, Duperron, et al., 2018b) were possibilities. Their presence was equally plausible based on the known distributions of these two species, because Vestnesa represents both the high Arctic setting of CPL‐clade members and the deep‐water environment of *O. haakonmosbiensis* (Figure 1).

According to the sequence results, *Oligobrachia haakonmosbiensis* is present at Vestnesa. This extends its distributional range to the high Arctic and shows it is not limited to the Norwegian Sea and that high latitude alone is not a barrier for dispersal and settlement. Instead, it appears to be a widespread species in northern regions, extending from the Nyegga/Storegga area in the Norwegian Sea (64°N) (Sen, Duperron, et al., 2018b; Smirnov, 2000) to the Fram Strait (79°N) (Figure 1). The specific sites that *O. haakonmosbiensis* is known from correspond exactly with northward flowing contour currents of Intermediate and Atlantic water (e.g., Hopkins, 1991). This strongly supports the idea of currents serving as the main dispersal mode for the species. Specifically, bottom currents are considered to transport Athecanephria frenulates (the group that refers to *Oligobrachia* and *Siboglinum* frenulates, among others). Members of this group supposedly lack a pelagic phase; the only phase at which transport away from adult populations is possible is when ciliated larvae are released after having been brooded within the maternal tube, and even then, there is a propensity for them to sink rapidly to the sediment (Bakke, 1974; Ivanov, 1963; Southward, 1999; Webb, 1964). Studies investigating frenulates across large geographic areas have demonstrated that species distributions tend to correlate with bottom currents (Hilário et al., 2010; Southward, 1972, 1971). Both brooding eggs and larvae have been observed for *O. haakonmosbiensis* (Sen, Duperron, et al., 2018b; Sen et al., 2019), which suggests that this species also lacks a pelagic phase. However, ciliated larvae have been observed swimming within maternal tubes and it is possible that larvae are capable of swimming in the external environment as well. It is unlikely that this swimming capacity is advanced or strong enough to prevent being swept along by currents. However, it could allow larvae to move vertically within the water column. The northward flowing surface and intermediate water masses, whose distribution *O. haakonmosbiensis* parallels, are contour currents (e.g. Hopkins, 1991). Whether *O. haakonmosbiensis* larvae are transported specifically by bottom water or flows higher up in the water column is, as a result, difficult to resolve.

The northward flowing currents likely responsible for the distribution of *O. haakonmosbiensis* also cross the western Barents Sea between mainland Norway and Svalbard (Figure 1). However, *O. haakonmosbiensis* is conspicuously absent from two known and studied seep sites on the Arctic shelf and Barents Sea at about 350 m water depth: the Storfjordrenna “pingo” site and the Bjørnøyrenna “crater” site (Sen, Duperron, et al., 2018b) (Figure 1). So far, *O. haakonmosbiensis* has only been found in relatively deep water on the continental slope between 750 m and 1,250 m (Decker et al., 2012; Gebruk et al., 2003; Sen et al., 2019; Smirnov, 2000). Therefore, in addition to currents, water depth probably represents another factor determining the distribution of *O. haakonmosbiensis*. No official boundary between deep and shallow waters exists; however, the distributions of seep siboglinids have previously been observed to be correlated with specific water depths. In the Gulf of Mexico, different species assemblages of seep vestimentiferans exist along a depth gradient, roughly at 1,000‐m‐depth intervals (Cowart, Halanych, Schaeffer, & Fisher, 2014; Miglietta, Hourdez, Cowart, Schaeffer, & Fisher, 2010). At seeps in general, species distributions and community characteristics tend to differ substantially above and below the 400–500 m water depth mark (Dando, 2010; Sahling et al., 2003; Sibuet & Olu, 1998). In the Gulf of Cadiz, the distribution of frenulate species was also seen to be determined to a certain extent by bathymetry (Hilário et al., 2010). The absence of *O, haakonmosbiensis* from shelf seep sites shallower than 400 m depth that are exposed to Atlantic water suggests it is a deep‐water species.

The other member of the cryptic *Oligobrachia* complex, the CPL‐clade (Sen, Duperron, et al., 2018b), was not present in our samples from Vestnesa, and it is possibly absent at this location. CPL‐clade members are, however, present at the two aforementioned high latitude Barents Sea seep sites of Storfjordrenna and Bjørnøyrenna at water depths of about 350 m, as well as the Laptev Sea at 60 m water depth (Figure 1, Sen, Duperron, et al., 2018b). *Oligobrachia* worms are also present at mud volcanoes in the Canadian Beaufort Sea (Paull et al., 2015). A recent study examined both 18S rRNA and mtCOI sequences of the Beaufort Sea individuals and demonstrated that mtCOI sequences align with those of *Oligobrachia* sp. CPL‐clade (99.6%–99.7% similarity, and forming a monophyletic clade with CPL‐clade members) (Lee et al., 2019). Nonetheless, the authors refrained from referring to Beaufort Sea *Oligobrachia* as CPL‐clade members since 18S rRNA sequences of the latter are not available for comparisons. However, 18S rRNA is a more conservative marker than mtCOI and less adept in discriminating between closely related species: Based on 18S rRNA sequences, the Beaufort Sea *Oligobrachia* are 99.9% similar to *O. haakonsmobiensis*, while based on mtCOI sequences, they are 96% similar to *O. haakonmosbiensis* (Lee et al., 2019). Beaufort Sea *Oligobrachia* thus ought to be considered members of the CPL‐clade as opposed to a distinct species as proposed by Lee et al. (2019). Their mtCOI sequence results support this, and no evidence currently exists suggesting that Beaufort Sea *Oligobrachia* are a distinct species from the CPL‐clade. We thereby consider *Oligobrachia* in the Beaufort Sea to be CPL‐clade members and refer to them as such. This means that *Oligobrachia* CPL‐clade is present in the Barents Sea, Laptev Sea, and the Beaufort Sea, at shelf and upper slope locations shallower than 500 m.

This distribution, combined with their absence from the deep‐water Arctic site of Vestnesa Ridge, suggests that the distribution of the CPL‐clade is also limited by depth, and the species inhabits shallower shelf and slope locations than *O. haakonmosbiensis*. Atlantic water likely transports the CPL species as it moves into the Barents and Laptev Seas; in these areas, though, cold, low‐salinity Polar water forms an overlying layer, with Atlantic‐derived water flowing as a subsurface current along the bottom (e.g., Bauch et al., 2009; Rudels, Jones, Schauer, & Eriksson, 2004). Therefore, similar to what has been considered for other frenulate species, CPL‐clade members might be transported primarily by bottom currents. This could additionally account for the presence of this species on the other (Canadian) side of the Arctic. Cooled, subsurface Atlantic water circulates the entire Arctic and finally exits through the western Fram Strait (e.g., Aagaard, 1981) (Figure 1). This circulation system around the Arctic likely serves as the dispersal method for the CPL‐clade and likely accounts for the pan‐Arctic distribution displayed by this species.

It should further be noted that the possibility still exists of the fjord species from northern Norway (69°N), *O. webbi* being the same as the CPL‐clade species (Sen, Duperron, et al., 2018b). It could, in fact be *O. haakonmosbiensis*, but given that its type locality is in shallow water, (*c*. 270 m water depth; Brattegard, 1966), the CPL‐clade would be the more likely candidate. However, that would mean that the CPL‐clade is not a high Arctic species. High latitude regions could thus have two distinct *Oligobrachia* species that are both distributed across the north Atlantic and the Arctic, where one is a deeper slope species and the other is a shallower shelf and slope species. Further studies and more extensive sampling are required to explore this notion.

### 4.2 New *Oligobrachia* species at Vestnesa

**Figure 8:**
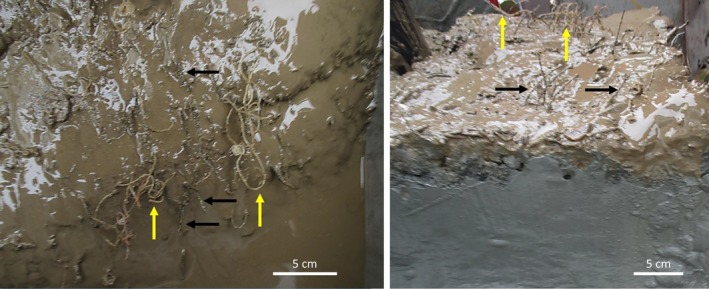
Box core sediments samples in which the two tube morphologies are visible, corresponding to the two collected frenulate species (left: top down view, right: side view). Yellow arrows indicate the new species with its light brown, curled tube, and black arrows show the darker brown, straight tubes of *Oligobrachia haakonmosbiensis*

The second type of sequence obtained in this study of Vestnesa frenulates is one that does not currently exist in the literature and potentially represents a new species altogether. The 3.86 ± 0.24% divergence in sequences, 19–20 mutation difference, and barcode gap all concur to indicate that these sequences represent a distinct species as opposed to being a subpopulation of either *O. haakonmosbiensis* or the CPL‐clade. Another possibility is that the new sequence represents *O. webbi* (Brattegard, 1966), that is virtually morphologically indistinguishable from members of the *Oligobrachia* clade (Sen, Duperron, et al., 2018b). Sequences do not exist for *O. webbi*; therefore, comparisons at the molecular level are not possible. However, there is a morphological line of evidence against the idea of the new sequence representing *O. webbi*. The tubes of mature individuals displaying the new sequence are all markedly distinct from *O. webbi* tubes and indeed from the tubes of other described *Oligobrachia* species. They tend to be lighter brown in color than the dark, almost black, brown color of other *Oligobrachia* species tubes (Figures 3, 4 and 8). More importantly, they were curled, particularly at the anterior ends, as opposed to being straight as other *Oligobrachia* species tubes. Species do display plasticity in terms of tube characteristics, and the CPL‐clade members from the Beaufort Sea even have curled tubes, albeit considerably different from the curled tubes of the new Vestnesa species (see figure 3c in Paull et al., 2015). Nonetheless, tube morphology is known to differ in frenulate species and can be used as a first morphological characteristic for distinguishing between species (Southward, 2000; Southward, Schulze, & Gardiner, 2005). Therefore, the distinctive curling and coloring of the second species in this study could indicate a new, previously unknown species. Unfortunately, detailed morphological examinations were not made; therefore, we are unable to make any conclusive statements on the morphology of the new species. Superficial examinations of individuals during sampling did not reveal any clearly discernable differences from *O. haakonmosbiensis* and the CPL‐clade. If the species represented by the new sequences is indeed a new species, it might add to the cryptic *Oligobrachia* species complex when one considers soft tissue structure and morphology, though the light brown, curled tube could be a distinct and representative feature of adults. Detailed and comprehensive taxonomic descriptions need to be carried out in order to appropriately reference this species within the scientific canon, but for the context of this study, we refer to it as the “new Vestnesa species.”

Other than the unusual, curled external tube, multiple individuals had a single tentacle, as opposed to the multiple tentacles usually seen in *Oligobrachia*. Only one frenulate genus, *Siboglinum*, is known to be unitentaculate (Ivanov, 1963; Southward, 2000; Southward et al., 2005). The sequencing results clearly demonstrate that the unitentaculate individuals at Vestnesa belong to the new species and are members of *Oligobrachia*, not *Siboglinum* (Figure 4). Though specific measurements were not possible onboard, overall, these individuals appeared to be thinner, that is, had smaller overall body diameters, than the multitentacled individuals of the new species, and individuals of *O. haakonmosbiensis*. Based on the limited number of live images taken and the remaining material available, a rough estimate indicates that single tentacle individuals were about 250–350 µm in diameter, while the multitentacled specimens had body and tube diameters between 500 and 930 µm (Figure 3, 4, 5). Their tubes were also distinctive in that they were seemingly very thin‐walled, colorless, and transparent such that the worms were easily visible within their tubes (Figure 5). The soft bodies of these individuals were also highly delicate. The extraction out of their tubes usually led to complete and immediate dissolution and forced us to leave the worms inside the tubes for the ensuing fixation and preservation. Additionally, during the DNA extraction process, they were retained within their tubes, but were nonetheless some of the easiest to digest (with Proteinase K as per the DNEasy kit protocol, which did not digest the other, more robust tubes of multitentaculate individuals of the new Vestnesa species and *O. haakonmosbiensis*).

Juvenile frenulates display a single tentacle and thin, colorless, transparent tubes (Ivanov, 1963; Southward, 1969). Although most adult frenulate species contain multiple tentacles, larvae develop one tentacle first, which appears initially as a tentacle bud (Ivanov, 1963). Only one detailed study on the development of late‐stage larvae of multitentacled frenulates exists. Southward (1969) examined the species *Polybrachia canadensis* and found that post settlement (in sediment) late‐stage larvae had only one well‐developed tentacle (adults have up to 40) and thin, colorless, and ringless tubes. Southward further mentions that juveniles of *Oligobrachia gracilis* have a unitentaculate juvenile stage that can be mistaken for *Siboglinum* (Southward, 1978). In fact, she cautions against assuming unitentaculate specimens as belonging to *Siboglinum* and urges using the tube, particularly if it is colorless and lacking the rings that are distinctive of adult tubes, as signifying the possibility of juveniles being present. Therefore, the single tentacle individuals of the new Vestnesa species are most likely juvenile individuals. Similar to our study, Hilário et al. (2010) recognized a unitentaculate specimen from the Gulf of Cadiz as not being *Siboglinum* based on mtCOI sequencing, thus emphasizing that combining morphological assessment with molecular methods is extremely important for species identification among frenulates and particularly among cryptic species such as those that inhabit north Atlantic and Arctic seeps.

Although thorough measurements were not made, all single tentacle individuals recovered for this study appeared to be similar in size and length and therefore at roughly the same developmental stage. Numerous single tentacle individuals were collected, and they all closely resembled each other in terms of overall size and thickness, although, regrettably, detailed measurements were not carried out. Since larvae likely sink and settle near adults (Bakke, 1974; Southward, 1975; Webb, 1964), the similarity in size could indicate that reproduction is synchronized, and on a large scale, across multiple pockmarks. Alternatively, it could indicate that reproduction is continuous, but recruitment and settlement in the sediment only takes place at certain times, when conditions are optimal. Though the length of time between fertilization and the development of the juvenile stage in *Oligobrachia* is unknown, Bakke's experiments on *S. fiordicum* (1974) revealed a lengthy larval phase with over a year passing before late larval stages with adult characteristics begin to develop (in this case, pinnules on tentacles). The juveniles observed in this study could have been the product of reproductive efforts by adults at least a year before our sampling took place. Other reproductive events might have occurred in the interim, but conditions might not have been adequate to allow settlement. Detailed studies are required to fully understand and adequately contextualize our observations, and in fact, the high abundance of juveniles in our samples means that Vestnesa represents an excellent choice for anyone wishing to study reproduction, larval development, and juvenile stages of frenulates.

### 4.3 Distribution and potential speciation events for the *Oligobrachia* clade

This work adds to the small body of molecular‐based research regarding high latitude seep frenulates (Lee et al., 2019; Sen, Duperron, et al., 2018b) and confirms the presence of three species across Nordic and Arctic waters. The confirmed presence of merely three species across such a large span of area is remarkable given that frenulate dispersal is considered likely to be quite slow overall (Southward & Southward, 1963; Southward, 1972, 1971). Large, yolk‐dependent eggs and nonplanktonic larvae, with a tendency of the latter to sink and burrow into the sediment, argue for most settlement to occur in the vicinity of adults (Bakke, 1974; Southward, 1972, 1971, 1969; Webb, 1964). Bottom currents would nonetheless carry propagules to a certain extent; however, the lack of a truly pelagic phase ought to restrict distributions. Slow dispersal rates are expected to result in multiple species over large geographic areas. Subsequently, in the Gulf of Mexico and the Caribbean, 17 species were identified based on morphology (Southward, 1972), and along the eastern coast of North America, this number goes up to 24 (Southward, 1971). Even when one considers only seep systems, the diversity across Nordic and Arctic sites is still very low. Among mud volcanoes in the Gulf of Cadiz, Hilário et al. (2010) used mtCOI sequencing to reveal 15 evolutionary lineages of frenulates. The distribution of a few species over large geographic areas in the north could demonstrate how well bottom currents can help in the dispersal of these completely nonpelagic animals (although as mentioned with *O. haakonmosbiensis*, dispersal might be aided by other currents if larvae are capable of vertical movement in the water column). The low diversity could additionally be restricted by conditions in high latitude regions, such as subzero ambient water temperatures (Åström et al., 2018; Portnova, Mokievsky, Haflidason, & Todt, 2014; Sen et al., 2019), or the strong seasonality in surface primary production that affects organic matter flux to the seafloor (since frenulates are capable of supplementing their symbiont‐derived diet with organic matter uptake from the sediment) (Dando et al., 2008; Southward & Southward, 1982; Southward, Southward, Brattegard, & Bakke, 1979). The presence of extensive grounded ice sheets during past glaciations could have additionally limited or affected the dispersal capabilities of many species. Furthermore, the low diversity of seep frenulates across northern regions could parallel the trend of overall low diversity in the Arctic. The widespread distributions of Arctic frenulates could represent dispersal through bottom water currents over lengthy periods of time.

Our data suggest a single speciation event between about 8 and 2 million years ago that led to the divergence of the three high latitude *Oligobrachia* species. It should be noted that this divergence date is not based on mutation rates for frenulates, but rather for hydrothermal vent species (Chevaldonné et al., 2002). Mutation rates for frenulates specifically do not currently exist, and we used mutation rates calculated for vent species since the vent environment is very similar to the seep environment in terms of energy sources and stressors, such as exposure to toxic chemicals such as hydrogen sulfide. Furthermore, siboglinids, even if not frenulates themselves, are included in this calculation. The divergence date range we suggest corresponds with the late Neogene, a period which was characterized by both uplift and accelerated subsidence in the Nordic seas and Fram Strait (e.g., Døssing et al., 2016; Japsen & Chalmers, 2000; Knies & Gaina, 2008). The resulting changes in the seafloor topography could have limited exchanges between populations or even isolation of some, which could have led to species being defined by bathymetry, in correspondence with present‐day distributions which appear to be constrained by water depth. Another important event during this period was the intensification of the North Atlantic thermohaline circulation. Exchange with Atlantic water started well before, with the opening of the Fram Strait around 17.5 Ma (Jakobsson et al., 2007) and variable overflows over the Greenland–Scotland Ridge beginning around 12 Ma (Poore, Samworth, White, Jones, & McCave, 2006). However, events that occurred later, such as deepening and stabilization of the Greenland–Scotland Ridge 6–2 Ma (Poore et al., 2006) and widening of the Fram Strait at 10 Ma, led to more steady and escalated water exchange as well as bottom current activity (Døssing et al., 2016; Kristoffersen, 1990). A later further deepening and widening of the Fram Strait occurred at 6–5 Ma, which caused additional intensification of the North Atlantic thermohaline circulation (Knies et al., 2014) and resulted in a highly stratified water column in the Nordic seas, as is seen today. This stratification, in conjunction with topographical changes, could have affected species radiation within northern *Oligobrachia*. Two of the sites where CPL‐clade members are present are located at the polar oceanic front where Polar and Atlantic surface water mix (Storfjordrenna and Bjørnoyrenna, Figure 1), which means this shelf species is exposed to fluctuating salinities as well as both negative and positive temperatures. Specialization for consistently low and usually negative temperatures (e.g., *O. haakonmosbiensis*) versus adaptations for varied temperature (and/or salinity) regimes (e.g., the CPL‐clade) could have therefore been a factor in the radiation of the northern *Oligobrachia* lineage. The late Neogene is also a time of general cooling and intensification of glacial–interglacial oscillations along with declining CO_2_ content of the atmosphere leading to the Northern hemisphere glaciations (Lisiecki & Raymo, 2005), which together with the closing of the Panama Isthmus from about 4 Ma (Driscoll, 1998) led to further intensification of the surface and deep water exchange between the North Atlantic and Arctic Ocean. This may have accelerated speciation rates of high latitude *Oligobrachia* worms.

## 5 CONCLUSIONS

We studied *Oligobrachia* species at Vestnesa pockmark methane seep sites and combined with previous work, we infer how northern seep frenulates are dispersed, which factors potentially control their distribution, and how these factors shed light on possible evolutionary and speciation events. Additionally, we demonstrate that juveniles are abundant at Vestnesa; therefore, this site could serve as a much needed source for examining reproduction in frenulates, a topic that has been somewhat difficult to pursue due to an overall lack of samples, particularly with respect to seep species. The relative simplicity of a few species with distributions that correlate well with oceanographic parameters means that northern latitude seeps could represent a natural laboratory for studying frenulates, a group of animals that despite potentially representing one of the most widespread symbioses in the deep sea (Rodrigues, Hilário, Cunha, Weightman, & Webster, 2011), has been studied only very modestly.

## CONFLICT OF INTEREST

The authors declare no competing interests.

## AUTHORS' CONTRIBUTIONS

AS conceived the study. TLR provided funds and ship time for sampling and oversaw activities at sea. AS conducted the sampling and shipboard processing. AS and AD conducted the laboratory work, and MMS provided access to laboratory space. SH carried out the bioinformatics analyses. AS wrote the main text of the article with major contributions from TLR and SH. All authors reviewed and approved the manuscript for submission.

## Data Availability

DNA sequences have been submitted to Genbank. Accession numbers are listed in Table 1. List of the individual worm samples used for this study
